# High-density SNP arrays improve detection of *HER2* amplification and polyploidy in breast tumors

**DOI:** 10.1186/s12885-015-1035-1

**Published:** 2015-02-06

**Authors:** Thomas v O Hansen, Jonas Vikesaa, Sine S Buhl, Henrik H Rossing, Vera Timmermans-Wielenga, Finn C Nielsen

**Affiliations:** 1 Center for Genomic Medicine, Copenhagen University Hospital, Blegdamsvej 9, DK-2100 Copenhagen, Denmark; 2 Department of Pathology, Rigshospitalet, Copenhagen University Hospital, Blegdamsvej 9, DK-2100 Copenhagen, Denmark

**Keywords:** Breast cancer, *HER2* amplification, Polyploidy, SNP array

## Abstract

**Background:**

Human epidermal growth factor receptor-2 (HER2) overexpression and gene amplification are currently established by immunohistochemistry (IHC) and fluorescence *in situ* hybridization (FISH), respectively. This study investigates whether high-density single nucleotide polymorphism (SNP) arrays can provide additional diagnostic power to assess *HER2* gene status.

**Methods:**

DNA from 65 breast tumor samples previously diagnosed by HER2 IHC and FISH analysis were blinded and examined for *HER2* copy number variation employing SNP array analysis.

**Results:**

SNP array analysis identified 24 (37%) samples with selective amplification or imbalance of the *HER2* region in the q-arm of chromosome 17. In contrast, only 15 (23%) tumors were found to have *HER2* amplification by IHC and FISH analysis. In total, there was a discrepancy in 19 (29%) samples between SNP array and IHC/FISH analysis. In 12 of these cases, the discrepancy towards FISH could be attributed to concomitant amplification or deletion of the centromeric region, which harbors the FISH reference probe sequence. In 3 tumors, repeated IHC/FISH analysis revealed that the original IHC/FISH analysis had failed to indicate the correct *HER2* expression level. Finally, the SNP array analysis revealed that more than two thirds of the samples exhibited polyploidy that was unrecognized by conventional FISH.

**Conclusions:**

Collectively, the data show that determination of *HER2* copy number variations by SNP array-based genomic segmentation analysis is an effective supplement to IHC/FISH *HER2* analysis that, by providing additional diagnostic sensitivity and accuracy, may elect more women for targeted treatment with HER2 inhibitors.

**Electronic supplementary material:**

The online version of this article (doi:10.1186/s12885-015-1035-1) contains supplementary material, which is available to authorized users.

## Background

Breast cancer is the most common type of cancer among women and approximately 430,000 new cases are diagnosed every year in Europe [1]. Breast cancer development and progression rely on several molecular pathways including estrogen receptor and human epidermal growth factor receptor-2 (HER2) receptor signaling, which represent important prognostic indicators and provide the molecular basis for targeted treatment by antibodies or small molecule inhibitors.

*HER2* is located on chromosome 17q12 and the gene is amplified in approximately 15–25% of breast cancers [2,3]. The HER/EGFR family of tyrosine kinases activates several mitogenic signaling pathways, such as the MAPK, PI3K/Akt, and mTOR pathways [4], and gene amplification is associated with a more aggressive course and reduced expression of estrogen and progesterone receptors [3,5,6]. On the other hand, antibodies or small molecule inhibitors such as Trastuzumab and Lapatinib efficiently inhibit the HER2 receptor and have been shown to improve overall survival and reduce risk of relapse [7-11]. Consequently, accurate testing of *HER2* amplification is of major importance for clinical decision-making in breast cancer patients.

*HER2* overexpression and gene amplification are normally established by a combination of immunohistochemistry (IHC) and fluorescence *in situ* hybridization (FISH). These procedures have been evaluated in a number of studies [12-19] and this has revealed that up to 20% of *HER2* testing results may be inaccurate [20]. Moreover, external quality assurance tests have indicated that due to the subjective nature of the IHC scoring system, fixation procedures, and histopathological assessments, only 75% of the participating laboratories consistently provided reproducible results [21,22]. As a result, a number of women are prevented from receiving the most effective treatment, while others are pointlessly treated with costly medicine with potentially harmful side effects [23]. As an illustration of the problem, it has been highlighted that a number of patients treated with Trastuzumab responded well [24,25] despite the fact that they tested negative for *HER2* amplification [26]. Finally, *HER2* FISH analysis has difficulties in identifying polysomy of chromosome 17 [27], which may complicate the interpretation of *HER2* testing results [28].

Thus, alternative methods are warranted to improve the accuracy of *HER2* analysis. Determination of copy number variations (CNVs) by high-density single nucleotide polymorphism (SNP) arrays is an appealing possibility because the technology provides an unbiased and highly reproducible measure of gene copy numbers. Furthermore, the analysis provides information about the entire genome, making it feasible to obtain data from other genes of interest, such as *TOP2A*, as they become validated for breast cancer diagnosis. In the present study, we assessed the *HER2* status of 65 breast tumors by high-density SNP array analysis and compared the results with those previously determined by IHC and FISH. Our data show that SNP arrays provide additional diagnostic sensitivity and accuracy compared to IHC and FISH analysis that appears to underestimate the number of cancers with *HER2* amplification. Therefore, SNP arrays could be a valuable supplement for analysis of *HER2* amplification by assigning more women with breast cancer to targeted treatment.

## Methods

### Patient samples

Breast cancer samples were routinely processed according to national guidelines. The original stainings and hybridizations were part of the routine clinical workload of the Department of Pathology, while SNP arrays were part of the routine analysis repertoire at the Center of Genomic Medicine. Therefore the scientific ethics committee of the Capital Region of Denmark determined that no ethical approval was necessary (H-3-2013-FSP55). The research was carried out in compliance with the Helsinki Declaration. Sixty-five breast cancer samples were collected between 2008 and 2009. The tumor samples were randomly selected comprising the following five categories: (1) IHC 0; (2) IHC 1+; (3) IHC 2+, not amplified by FISH; (4) IHC 2+, amplified by FISH; and (5) IHC 3+. The study aimed to contain 50% HER 2+ tumor samples.

### Immunohistochemistry (IHC)

Fresh breast tumor tissue was immediately placed in formalin fixative and paraffin embedded. Six-micrometer sections were cut from the tissue blocks and mounted on Super Frost Plus slides (Menzel-Gläser). *HER2* status was assessed using the HercepTest™ kit K5207 (Dako) and HercepTest™ Autostainer plus link (Dako) following the manufacturer’s recommendations. Processed immunohistochemical slides were scored according to the recommendations of the American Society of Clinical Oncology/College of American Pathologists [20]. Each case was categorized as 0, 1+, 2+ or 3+ and specimens scoring 3+ were considered as *HER2* positive. Cases scoring 2+ were regarded as equivocal and were subsequently assessed by *HER2* FISH analysis. Cases scored as 0 or 1+ were considered as *HER2* negative. All original stainings were part of the routine clinical workload of the Department of Pathology and the results were acquired from the clinical records and therefore represent the actual readings of different pathologists. The analysis is subjected to both internal and external control (UK NEQAS).

### Fluorescence *in situ* hybridization (FISH)

FISH was performed on all cases scoring 2+ on HercepTest™ and for quality control purposes on a proportion of the divergent cases from the study group. Sections of 2–4-μm thickness were cut from paraffin blocks, mounted on Super Frost Plus slides and baked for 60 min at 60°C. HER2 status was assessed using the HER2 FISH pharmDx™ kit K5331 (Dako). The HER2 probes were labeled with Tx-Red and the control probe mix targeting the centromere on chromosome 17 (CEP17) was labeled with FITC. The level of HER2 gene amplification was determined in the tissue sections by counting the green and red signals in the nuclei of a minimum of 20 invasive carcinoma cells. The amplification ratio is the ratio of red to green signals in each section, using a cut-off point of 2. Cases with a ratio of 2 or more were regarded to have amplification of the HER2 gene. All original hybridizations were part of the routine clinical workload of the Department of Pathology and the results were acquired from the clinical records and therefore represent the actual readings of different pathologists. The analysis is subjected to both internal and external control (UK NEQAS).

In order to assess FISH staining per nucleus, the standard protocol was modified as follows. Paraffin section thickness was increased to 10–12 μm. Labeling was performed as described above, except TO-PRO3 (Invitrogen) was applied as a nuclear marker. Sections were examined on a Zeiss LSM 510 Confocal Microscope, using a 100× objective and a multitrack triple color setting. Consecutive confocal images were taken with a thickness of 0.39 μm. Three-dimensional models were generated from the Z-stack sections using the Zeiss Image browser software. A minimum of two Z-stacks containing >30 cells were generated per sample. The results are stated as the observed average of HER2 copies per nucleus.

### DNA purification

DNA was purified from snap-frozen breast cancer samples macrodissected by a pathologist. The samples were incubated at 55°C overnight in 200 μl TNES buffer (10 mM Tris–HCl (pH 7.5), 400 mM NaCl, 100 mM EDTA, 0.6% SDS) and 20 μl Proteinase K (20 mg/ml). Genomic DNA was isolated using NaCl precipitation, washed with 70% ethanol, dried and resuspended in Tris-EDTA buffer. The DNA integrity was examined by agarose gel analysis and the DNA concentration was determined using the NanoDrop ND-1000 spectrophotometer (NanoDrop Technologies).

### SNP array analysis

Forty-seven DNA samples were analyzed using Affymetrix SNP 500 K arrays and 18 DNA samples were analyzed using the SNP 6.0 array according to Affymetrix’s instructions. CEL files were analyzed with Partek Genomics Suite 6.5. Data were imported using the default Partek settings, including adjustments for probe sequence, background and quantile normalization, and allele-specific summarizing of probes. Copy number state and SNP allele ratio were calculated by an unpaired analysis using a baseline generated from 76 healthy Danish controls in the case of the SNP 500 K, and 270 samples from the international HapMap project for the SNP 6.0 arrays. The average value of all copy number probe intensity calls across the genome was assigned as copy number state 2. Segmentation analysis was performed on copy number probe intensity calls using Partek’s genomic segmentation algorithm, which determines breakpoints in the data rather than calculating a predefined copy number state. The algorithm determines a segment using the following criteria: (1) neighboring regions have statistically different average intensities (p < 0.001), (2) breakpoints are chosen to give the best statistical significance (smallest p-value), (3) detected regions must contain a minimum number of data points (SNP 500 K = 100, SNP 6.0 = 200), and (4) the minimum magnitude of changes to be detected relative to the noise estimate for each chromosome is set to 0.3. The detected segments were analyzed in order to determine their copy number status (normal, deletion or amplification). The copy number range was set to 0.2 and the p-value threshold to 0.01, so <1.8 = deletion, 1.8 < × < 2.2 = normal, >2.2 = amplification. In order to verify that our genomic segmentation model parameters did indeed call actual strand breaks, we conducted a visual inspection of predicted strand breaks verifying a change in allele ratio. This showed that the 1.8 < × <2.2 segmentation model provides a conservative estimate with no false-positive strand breaks.

To determine the extent of polyploidy in the tumor samples, the different copy number fragment states in combination with the allelic balances at all chromosomes was examined. A tumor was assigned as polyploid if the median intensity probes throughout all chromosomes were concluded to be 3 copies or more. It was not always possible to determine the precise number of the polyploid state, and in these cases only the lowest possible estimate is indicated.

## Results

### Pathological characteristics of the breast tumor samples

Sixty-five primary breast tumor samples were selected for the study, comprising 12 tumors scored as IHC 0, 15 as IHC 1+, 32 as IHC 2+, and 6 as IHC 3+ (Table 1, HER2 IHC). The *HER2*/CEP17 ratios of all the IHC 2+ tumor samples were examined by FISH analysis (Table 1, HER2 FISH). Nine of the IHC 2+ tumors had a *HER2*/CEP17 ratio of more than 2.0, 18 IHC 2+ tumor samples had a *HER2*/CEP17 ratio between 1.5 and 2.0, while the remaining 5 IHC 2+ tumor samples had a *HER2*/CEP17 ratio below 1.5. In total, 15 (23%) tumor samples were found to have *HER2* amplification by IHC and FISH analysis. Other pathological data is shown in Additional file 1.

**Table 1:** **Comparison of IHC, FISH and SNP data**

	IHC/FISH		SNP array		
Tumor sample number	HER2 IHC	HER2 FISH	HER2 segment copy number	HER2/CEP17 ratio	HER2 copy number status
1	0	n/a	1,98	balance	normal
2	0	n/a	2,35	HER2+	amp*
3	0	1,92	1,81	balance	normal
4	0	n/a	2,06	balance	normal
5	0	n/a	2,04	balance	normal
6	0	n/a	1,72	cent+	del
7	0	n/a	1,67	balance	del
8	0	n/a	1,83	balance	normal
9	0	n/a	1,51	cent+	del
10	0	n/a	2,51	balance	amp*
11	0	n/a	1,78	balance	del
12	0	n/a	1,85	balance	normal
13	1	n/a	1,97	balance	normal
14	1	n/a	2,08	balance	normal
15	1	0,83	2,33	balance	amp*
16	1	0,80	1,70	balance	del
17	1	0,80	1,80	cent+	normal
18	1	0,86	2,49	balance	amp*
19	1	n/a	2,06	balance	normal
20	1	n/a	2,09	balance	normal
21	1	n/a	1,96	balance	normal
22	1	n/a	1,97	balance	normal
23	1	n/a	2,13	balance	normal
24	1	n/a	1,90	balance	normal
25	1	n/a	1,87	balance	normal
26	1	n/a	4,46	balance	amp*
27	1	n/a	2,34	balance	amp*
28	2	1,41	2,45	balance	amp*
29	2	1,54	2,16	balance	normal
30	2	3,05*	2,04	balance	normal
31	2	2,69*	2,48	balance	amp*
32	2	2,42*	1,75	cent+	del
33	2	1,73	1,99	balance	normal
34	2	2,65*	1,92	balance	normal
35	2	1,50	1,91	balance	normal
36	2	1,57	2,00	balance	normal
37	2	1,68	1,79	balance	del
38	2	1,90	1,84	balance	normal
39	2	1,60	2,05	balance	normal
40	2	1,58	1,89	balance	normal
41	2	2,13*	2,73	balance	amp*
42	2	1,54	2,04	balance	normal
43	2	1,82	1,54	balance	del
44	2	1,90	1,77	balance	del
45	2	1,66	2,33	balance	amp*
46	2	1,48	2,04	balance	normal
47	2	1,73	1,98	balance	normal
48	2	1,67	2,36	balance	amp*
49	**2/3**	**0,93/8,8**	11,10	HER2+	amp*
50	2	1,81	2,04	balance	normal
51	2	1,58	2,65	HER2+	amp*
52	2	1,60	2,23	balance	amp*
53	2	2,27*	8,03	HER2+	amp*
54	2	1,87	2,91	balance	amp*
55	2	1,24	2,66	balance	amp*
56	2	7,38*	7,01	HER2+	amp*
57	2	1,00	1,88	balance	normal
58	2	8,60*	7,98	HER2+	amp*
59	2	3,24*	3,18	HER2+	amp*
60	3*/1	**1,65**	2,00	balance	normal
61	3	4,11*	5,28	HER2+	amp*
62	3	7,00*	4,58	HER2+	amp*
63	3*/2	**1,50**	2,05	balance	normal
64	3*	n/a	3,15	HER2+	amp*
65	3*	1,35	3,75	HER2+	amp*

Asterisk indicates *HER2* positive results, while bold indicates samples that have been reanalyzed. *Abbreviations*: *amp* amplified *CEP17* centromere on chromosome 17, *del* deletion, *FISH* fluorescent in situ hybridization, *HER2* human epidermal growth factor receptor-2, *IHC* immunohistochemistry, *n/a* not analysed.

### SNP array copy number analysis

Sixty-five frozen tumors were initially examined by SNP 500 K or SNP 6.0 arrays in a blinded manner. To examine the reproducibility of the SNP assay, 10 tumor samples were initially replicated on newly isolated DNA using SNP 500 K. All the replicated samples exhibited identical results. Moreover, CNVs detected by the separate 500 K *Sty*I and *Nsp*I arrays were in all cases completely overlapping, inferring that the technical variation in the array-based detection of CNVs was negligible.

Copy number variations and breakpoints were depicted and the compiled results of all chromosomes from all tumor samples are shown in Additional file 2. The tumors exhibited a large number of different CNVs across the whole genome, in agreement with previous results [29,30].

**Figure 1:**
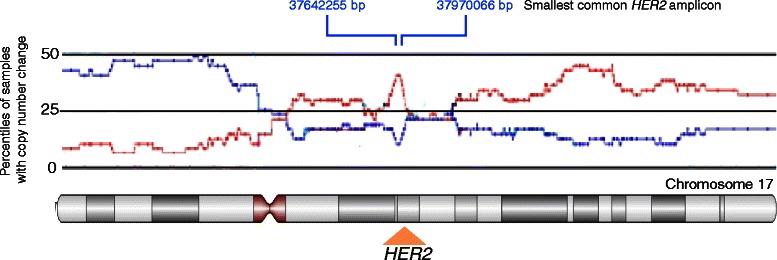
**Schematic representation of copy number variations across chromosome 17.** The percentiles of SNP samples with amplification or deletion among the breast tumor samples analysed on SNP 500 K are indicated in red and blue, respectively. The positions of *HER2* and the smallest amplicon containing 9 full-length genes (from 37642255 to 37970066 based on Hg19) are indicated.

According to the genomic segmentation analysis, 24 (37%) tumor samples exhibited selective amplification of the *HER2* region, whereas 9 had *HER2* deletion and 32 exhibited normal *HER2* status (Table 1, HER2 copy number status). Four-fold or greater amplification (4.46–11.10-fold) of *HER2* was observed in 7 samples (tumor sample 26, 49, 53, 56, 58, 61, and 62), whereas 17 samples (tumor sample 2, 10, 15, 18, 27, 28, 31, 41, 45, 48, 51, 52, 54, 55, 59, 64, and 65) had a more moderate *HER2* amplification (2.23–3.75-fold). The minimal amplified region surrounding *HER2* according to the genomic segmentation algorithm covered ~328 kb DNA (Figure 1), while the largest amplified region consisted of a complete duplication of chromosome 17 (in two cases). The minimal region contained 11 genes that – in addition to *HER2* – comprised partial *CDK12*, *NEUROD2*, *PPP1R1B*, *STARD3*, *TCAD*, *PNMT*, *PGAP3*, *c17orf37*, *GRB7*, partial *IKZF3*.

### Comparison of SNP array data with IHC and FISH

**Figure 2:**
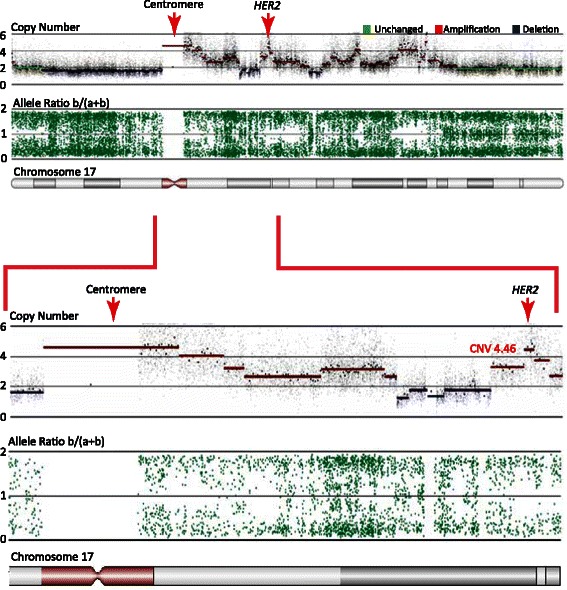
**Co-amplification of chromosome 17 centromere and*HER2*.** SNP data from tumor sample 26 displaying the copy number and allele ratios across chromosome 17. This sample has undergone multiple DNA breaks and several regions of the chromosome 17q arm are amplified, including a region around *HER2* (4.46-fold) and a region close to the centromere. The two regions are amplified to the same extent and may therefore explain the negative IHC *HER2*/CEP17 even though the *HER2* gene is clearly amplified.

In 46 (71%) tumor samples, the *HER2* status was confirmed by SNP array analysis (Table 1). In 19 (29%) tumor samples, however, there was a discrepancy between the SNP analysis and IHC/FISH, including 2/12 (16.7%) of the IHC 0 samples, 4/15 (26.7%) of the IHC 1+ samples, 11/32 (34.4%) of the IHC 2+ samples, and 2/6 (33.3%) of the IHC 3+ samples. Altogether, the 19 samples comprised 14 IHC/FISH-negative and 5 IHC/FISH-positive *HER2* results. According to the SNP array data, 11 of the IHC/FISH-negative samples (tumor sample 10, 15, 18, 26, 27, 28, 45, 48, 52, 54, and 55) exhibited *HER2* amplification without any change in the *HER2*/CEP17 ratio. Tumor sample 26 is shown in Figure 2 as an example. One sample (tumor sample 49) was reanalyzed by IHC/FISH, and this analysis revised the classification, previously scored as IHC 2+ with a *HER2*/CEP17 ratio of 0.93, to IHC 3+ with a *HER2*/CEP17 ratio of 8.8 (Table 1). The last 2 IHC/FISH-negative samples were scored as IHC 0 (tumor sample 2) and IHC 2+ (tumor sample 51) with a *HER2*/CEP17 ratio of 1.58. Both tumor samples had a moderate but visible *HER2* amplification when analyzed by SNP array (Table 1). Among the IHC/FISH-positive *HER2* samples, 2 were IHC 3+ cases (tumor sample 60 and 63). These 2 cases were reanalyzed by IHC and FISH, and were subsequently reclassified as IHC 1+ and IHC 2+, respectively, with a *HER2*/CEP17 ratio of 1.65 and 1.5, respectively. The 3 other IHC/FISH-positive samples were all scored as IHC 2+ (tumor sample 30, 32, and 34) with a *HER2*/CEP17 ratio of 3.05, 2.42, and 2.65, respectively. In these cases, the SNP array data classified the tumor samples as normal (tumor sample 30 and 34) or with *HER2* deletion (tumor sample 32).

### Polyploidy

**Figure 3:**
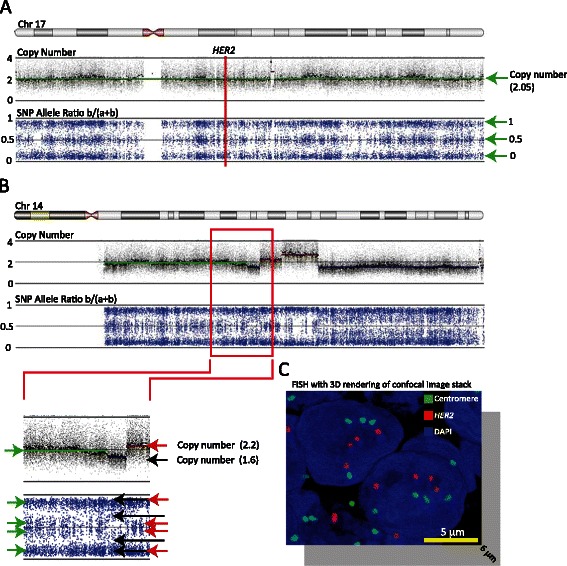
**Detection of polyploidy. (A)** SNP and copy number data across chromosome 17 from tumor sample 5. The top panel displays the copy number probe intensity calls and the calculated copy number segments (in color). The calculated segment (green line) has an intensity value of just over 2. The lower panel displays the calculated SNP allele ratios and shows that the entire chromosome 17 is in allelic balance. The vertical red line indicates the position of *HER2*. **(B)** SNP and copy number data across chromosome 14 from tumor sample 5. The enlargement of the red box shows that a segment (green line) is predicted with an intensity value of just under 2. However, a weak allelic imbalance (green arrows) suggests that the intensity value of just under 2 does not correspond to 2 DNA copies. Moreover, a deletion (~1.6) and an amplification (~2.2) only result in a modest copy number intensity change. Taken together, the data in **(A)** and **(B)** suggest that a segment with an intensity value of just over 2 and allelic balance must correspond to at least 4 copies of DNA. **(C)** Representative image of a 3D-rendered model of a confocal image stack of a section from tumor sample 5 hybridized with *HER2* (red) and CEP17 (green) probes. The image extends 6 μm down into the z-axis, corresponding to ~60–70% of the nucleus diameter.

During the analysis of the SNP array data, it became evident that the baseline 2 value was incorrectly mapped in a number of samples because the SNP probes indicated imbalance despite the fact that copy numbers were centered around 2. By combining the information of copy number fragment states and allelic balances, it was possible to establish that the samples were in fact polyploid (defined as having more than 2 sets of all chromosomes). As an example, tumor sample 5 (Figure 3) did not exhibit any local CNVs in chromosome 17. However, on chromosome 14, it could be deducted that the assigned copy number state 2 intensity value corresponded to 4 DNA copies (for detailed information, please refer to the figure legend). Additional file 3 shows an additional case of polyploidy in tumor sample 45. In total, 45 (69%) of the tumor samples were found to have undergone global duplication of their entire genome (Table 2). The polyploid status was correlated to *HER2* copy number (Table 2, HER2 copy number estimate). To verify the calling of polyploidy, we modified the *HER2* FISH protocol and increased the section thickness to 10–12 μm in order to encompass a whole nucleus. The sections were examined by confocal microscopy with high magnification and Z-stack imaging generated 3D-rendered reconstructions of the entire nucleus. Nine samples that were assigned as being polyploid were examined. Since cells in late S or G2 phase will display double the amount of *HER2* and CEP17 probe signals, these cells were excluded from the analysis. The number of *HER2* gene copies (red dots) observed per nucleus/tumor cell is listed in Table 2 (*HER2* copy number per nucleus (3D FISH)). In all nine cases, the analysis confirmed that the tumor samples were polyploid. Representative images of the 3D renderings are shown in Figure 3C and Additional file 3. In both cases, more than 2 copies of both *HER2* and CEP17 were found to exist. Moreover, in all nine tumor samples, the observed number of copies of *HER2* (red signal) and CEP17 (green signal) was found to be in accordance with the estimated copy number based on SNP array calculations alone. This finding was confirmed using probes recognizing the centromere of chromosome 7 (CEP7) and chromosome 17 (CEP17), respectively (data not shown).

**Table 2:** **Summary of polyploidy**

Sample number	Polyploidy	HER2 copy number estimate	HER2 copy number per nucleus (3D FISH)
1	No	normal	
2	Yes	5+	
3	Yes	3+	
4	No	normal	
5	Yes	4+	4-5
6	Yes	3+	
7	Yes	3+	
8	Yes	4+	
9	Yes	3+	
10	No	3	
11	Yes	3+	
12	Yes	5+	
13	No	normal	
14	No	normal	
15	Yes	5+	
16	Yes	3+	
17	Yes	3+	
18	No	3+	
19	No	normal	
20	No	normal	
21	Yes	5+	
22	Yes	4	
23	Yes	4+	
24	Yes	3+	
25	No	normal	
26	Yes	6+	
27	Yes	4+	5-7
28	Yes	4+	
29	Yes	4+	4-6
30	Yes	5+	
31	Yes	5+	5-7
32	Yes	3+	
33	No	normal	
34	Yes	5+	5-7
35	No	normal	
36	Yes	5+	4-5
37	Yes	3+	
38	Yes	3+	
39	No	normal	
40	No	normal	
41	Yes	5+	
42	No	normal	
43	Yes	UPD(2) or amp3	2
44	Yes	5+	
45	Yes	8+	8
46	Yes	4+	
47	No	normal	
48	Yes	5+	
49	Yes	25+	
50	Yes	5+	
51	No	3	
52	No	3	
53	Yes	7+	
54	Yes	7+	
55	Yes	7+	5-6
56	Yes	12+	
57	No	normal	
58	Yes	12+	
59	Yes	6+	
60	No	normal	
61	Yes	8+	
62	Yes	8+	
63	No	normal	
64	Yes	7+	
65	Yes	9+	

*Abbreviations*: *FISH* fluorescent in situ hybridization, *HER2* human epidermal growth factor receptor-2, *UPD* uniparental disomy.

## Discussion

According to the guidelines of the American Society of Clinical Oncology (ASCO)/College of American Pathologists (CAP), *HER2* overexpression and gene amplification should be established by a combination of IHC and FISH. Recent reports indicate, however, that about 20% of *HER2* testing results may be incorrect [20]. Moreover, in a group of HER2 IHC 0 tumor samples, it has been reported that FISH identified positive *HER2* amplification in 2–8% of the cases, while 5–22% of HER2 IHC 3+ tumor samples were found to lack *HER2* amplification according to FISH (reviewed in [31]). Since the correct measurement of *HER2* copy numbers is essential for instigation of targeted therapy [32,33], we investigated whether it is possible to improve the accuracy of the analysis by employing high-density SNP arrays.

SNP arrays were developed for global analysis of single nucleotide polymorphisms, but by adding information about the intensity of particular SNPs, the analysis may be exploited to identify CNVs with high sensitivity, resolution, and reproducibility [34,35]. Current high-density SNP arrays detect CNVs in the range of about 5–10 kb. Considering that the *HER2* amplicon covers at least 280 kb DNA [36,37], the resolution of SNP-based CNV detection is more than sufficient to provide detailed information about the amplicon. Moreover SNP arrays are able to detect alterations in samples containing down to 10% tumor tissue [38].

Several different algorithms based on simple defined thresholds to complex statistical modeling have been developed to call CNVs. We employed a genomic segmentation algorithm which defines breakpoints based on systematic change in intensity along the chromosome, unlike the Hidden Markov Model which allocates intensities to a predefined copy number state (0, 1, 2, 3……). In this way, the segmentation algorithm allows the identification of CNVs that are less than a whole copy number state. We considered this to be important for clinical use because factors such as contamination by normal tissue, tumor heterogeneity, and polyploidy in the tumor may lead to fractional copy number changes.

The discrepancy between SNP arrays and FISH can be mainly explained by the fact that 12 amplicons included the centromeric region of chromosome 17 that harbors the binding site of the CEP17 reference probe. CEP17 may therefore not be the optimal reference probe and other centromere probes, e.g. on chromosome 2 or 9, which comprise few rearrangements in breast tumors are recommended (Additional file 2). In 3 samples, revision of the IHC/FISH analysis showed that they were in fact in agreement with the SNP data. Taken together, the results indicate that SNP arrays increase the sensitivity and specificity of the *HER2* analysis.

In addition, SNP arrays provide information about polyploidy, which is generally considered to reflect genomic instability and may promote cell transformation [39]. Increased *HER2* gene copy number caused by chromosome 17 polysomy has been reported to be a contributing factor in HER2 overexpression in otherwise unamplified invasive breast carcinomas [40]. It was proposed that cases carrying chromosome 17 polysomy should be further evaluated for HER2 protein overexpression by IHC. Other studies, however, have failed to demonstrate a correlation between chromosome 17 polysomy and expression of HER2 protein [28,41,42].

We observed that more than two thirds of the tumors studied here were polyploidy, including 18 polyploid samples determined as IHC 0 or IHC 1+. Although our tumor material was selected to contain a large number of IHC 2+ tumors, the data are in contrast to recent studies reporting chromosome 17 polysomy (defined as 3 or more copies of the chromosome 17 centromere) with a frequency of 0–46% [27,28,41,43-53]. Comparative genomic hybridization (CGH) array studies in particular have concluded that chromosome 17 polysomy in breast cancer is rare and only occurs in about 5% of tumors [27,52]. CGH array may, however, not be optimal for detection of polyploidy/chromosome 17 polysomy because the data analysis is based on the Hidden Markov Model which may fail to uncover polyploidy. Further studies are required to clarify the significance of chromosome 17 polysomy in breast cancer patients.

Other techniques of HER2 testing besides IHC and FISH analysis have previously been suggested, including chromogenic *in situ* hybridisation (CISH) [54,55], silver enhanced *in situ* hybridisation (SISH) [56], Q-RT-PCR [57] and multiplex ligation-dependent probe amplification (MLPA) [58]. Our study shows that SNP array should be included as a HER2 testing method as well. Moreover, recent changes in clinical protocols require examination of deletions and amplification of the *TOP2A* gene as well. In the near term future we expect a rising demand in the examination of several gene alterations from the same tumor. In this regard SNP chip array will be a time and money saving procedure investigating these genomic alterations, since it includes the simultaneous examination of all chromosomes. Moreover, SNP arrays can be analyzed within three working days, so results can be provided to the clinical department in the same time frame as IHC/FISH data. One limitation for the clinical use of SNP arrays is the use of fresh-frozen tumor tissue, since fresh-frozen tumor samples are not available for routine diagnostics in many countries. However protocols for SNP arrays using formalin-fixed paraffin-embedded tissue have recently been described [59], suggesting a broader application of SNP array analysis in a clinical setting in the near future.

## Conclusion

In summary, we conclude that copy number analysis by means of SNP arrays offers a number of advantages and improvements that may warrant their use in *HER2* diagnostics. Most importantly, array-based analysis is accurate and identifies more breast tumors for targeted treatment. The analysis is fast and generates highly reproducible and quantitative data. Moreover, it provides a global view that allows rapid evaluation of multiple regions of interest. Future studies are needed to evaluate response of Trastuzumab in patients with HER2 positive tumors identified by SNP array analysis. This could be done retrospective or in a randomised control trial.

## Additional files

Additional file 1: Table S1. Pathological data of 65 breast tumors. The breast tumors consisted of ductal carcinomas, lobular carcinomas, mixed ductal/lobular carcinomas, or mucinous carcinomas. The tumors were graded according to the following: Grading 3–5 = Grade 1, 6–7 = Grade 2, 8–9 = Grade 3. The mucinous tumors were not graded. ER and PgR were regarded as negative (0) when staining is less than 10%. One tumor (29) was of unknown ER and PgR status. Abbreviations: DC, ductal carcinoma; Diam, diameter; ER, estrogen receptor; LC, lobular cancinoma; MC: mucinous carcinoma; PgR, progesterone receptor.
Additional file 2: Figure S1. Copy number variations observed in the 65 breast tumors showing the frequencies of genomic copy number gains and losses plotted according to their genomic localization. Blue lines correspond to allelic losses and red lines depict gains. Data from SNP 500 K and SNP 6.0 are displayed separately. Sixty percent of the tumors have amplification of chromosome 1q. A third of the samples have amplification of chromosome 5p, while about half of the tumors have loss of chromosome 8p from p.12 and beyond – frequently in combination with amplification of chromosome 8q, identified in about 60% of the tumors. Almost a third of the tumors show a high copy number amplification of the end of chromosome 8 from p.11.21 into the beginning of p.12 and approximately half of the tumors have loss of chromosome 11q from q.14.1 to the telomere. Finally, amplification of chromosome 16p is seen in almost half of the tumors and often in combination with loss of chromosome 16q, while loss of chromosome 17p is seen in about 40% of the tumors.
Additional file 3: Figure S2. Detection of polyploidy. **(A)** SNP and copy number data across chromosome 3 from tumor sample 45. The top panel displays the copy number probe intensity calls and the calculated copy number segments (in color). The lower panel displays the calculated SNP allele ratios of chromosome 3. The calculated segments have varying intensity values. The fragment with the lowest intensity value represents at least 3 copies because it exhibits allelic imbalance but still displays SNP heterozygosity (red arrows). Each fragment can be assigned an increasing copy number intensity, revealing that the predicted ‘copy number 2’ intensity corresponds to between 5 and 6 copies of DNA. **(B)** Subsequent examination of chromosome 17 shows that *HER2* must be present in at least 7 copies. This sample also displays amplification of the centromere region (q-arm side) to the same extent as *HER2*, explaining why the FISH *HER2/*CEP17 ratio is 1.66. The vertical red line indicates the position of the centromere and *HER2*. **(C)** Representative image of a 3D-rendered model of a confocal image stack of a section from tumor sample 45 hybridized with *HER2* (red) and CEP17 (green) probes. The image extends 6 μm down into the z-axis, corresponding to ~60–70% of the nucleus diameter.
