# Supplementary material for: High-density SNP arrays improve detection of HER2 amplification and polyploidy in breast tumors
Source: BMC Cancer. 2015 Feb 6;15:35. doi: 10.1186/s12885-015-1035-1 (PMC4326399; doi:10.1186/s12885-015-1035-1)
Supplement: Additional file 1: Table S1. — Pathological data of 65 breast tumors. The breast tumors consisted of ductal carcinomas, lobular carcinomas, mixed ductal/lobular carcinomas, or mucinous carcinomas. The tumors were graded according to the following: Grading 3–5 = Grade 1, 6–7 = Grade 2, 8–9 = Grade 3. The mucinous tumors were not graded. ER and PgR were regarded as negative (0) when staining is less than 10%. One tumor (29) was of unknown ER and PgR status. Abbreviations: DC, ductal carcinoma; Diam, diameter; ER, estrogen receptor; LC, lobular cancinoma; MC: mucinous carcinoma; PgR, progesterone receptor. [file 12885_2015_1035_MOESM1_ESM.pdf]

Sample number	Type	Grade	Grading	Diam	ER	PgR
1	LC	2	3+1+2	40	100	100
2	DC	3	3+3+3	60	0	0
3	LC	2	3+1+1	73	0	0
4	DC	2	3+1+2	11	100	20
5	DC	1	2+1+2	15	100	100
6	DC	3	3+3+3	19	0	0
7	DC	3	3+3+3	60	0	0
8	DC	3	2+3+3	19	0	0
9	DC	3	3+3+3	24	0	0
10	DC	2	2+1+3	25	100	90
11	DC	3	3+3+3	25	0	0
12	LC	2	3+1+2	22	100	80
13	DC	1	2+1+2	18	70	100
14	LC	2	3+1+2	21	80	20
15	DC	3	3+2+3	21	100	100
16	LC	2	3+1+2	20	0	0
17	DC	3	3+2+3	20	0	0
18	DC	2	3+2+2	40	50	100
19	DC	2	3+1+2	25	80	0
20	LC	2	3+1+2	14	100	0
21	DC	3	3+2+3	14	40	10
22	DC	2	2+2+3	23	100	90
23	DC/LC	2	3+2+2	21	95	100
24	DC	2	3+1+2	30	100	50
25	DC	2	3+2+2	22	100	100
26	DC	3	3+3+3	40	100	90
27	DC	1	2+1+2	25	80	20
28	DC	3	3+3+3	50	0	0
29	DC	2	3+1+2	22	80	80
30	DC	3	3+2+3	23	60	20
31	DC	3	3+3+3	30	100	0
32	DC	3	3+3+3	40	100	90
33	DC	2	3+1+2	12	100	100
34	DC	3	3+3+2	21	60	0
35	DC	3	3+3+2	15	90	60
36	DC	2	3+2+2	30	99	90
37	DC	3	2+3+3	18	0	0
38	DC	3	3+2+3	15	0	0
39	MC			35	100	90
40	DC	2	2+2+2	12	99	99
41	DC	3	3+2+3	21	100	100
42	DC	2	2+2+3	15	50	40
43	DC	3	3+3+3	21	0	0
44	DC	3	3+3+3	22	0	0
45	DC	3	3+2+3	25	0	0
46	LC	2	3+1+2	21	90	100

47	DC	2	2+2+2	18	100	100
48	DC	2	2+2+2	12	100	80
49	MC			12	60	10
50	LC	2	3+1+2	35	100	20
51	DC	3	3+3+3	39	100	0
52	DC	2	3+2+2	18	100	100
53	DC	2	3+2+2	38	100	0
54	DC	2	3+1+3	21	0	0
55	DC	2	3+1+2	100	100	15
56	DC	2	3+1+3	30	90	90
57	DC	2	3+1+2	22	90	0
58	DC	3	3+3+3	27	0	0
59	DC	2	3+1+2	21	0	0
60	DC	2	3+2+2	17	100	90
61	DC	3	3+3+3	50	0	0
62	DC	3	3+3+3	65	0	0
63	DC	2	3+1+2	21	50	80
64	DC	3	2+3+3	22	90	0
65	DC	3	3+3+3	32	0	0
