# Supplementary figures and images for: High-density SNP arrays improve detection of HER2 amplification and polyploidy in breast tumors
Source: BMC Cancer. 2015 Feb 6;15:35. doi: 10.1186/s12885-015-1035-1 (PMC4326399; doi:10.1186/s12885-015-1035-1)

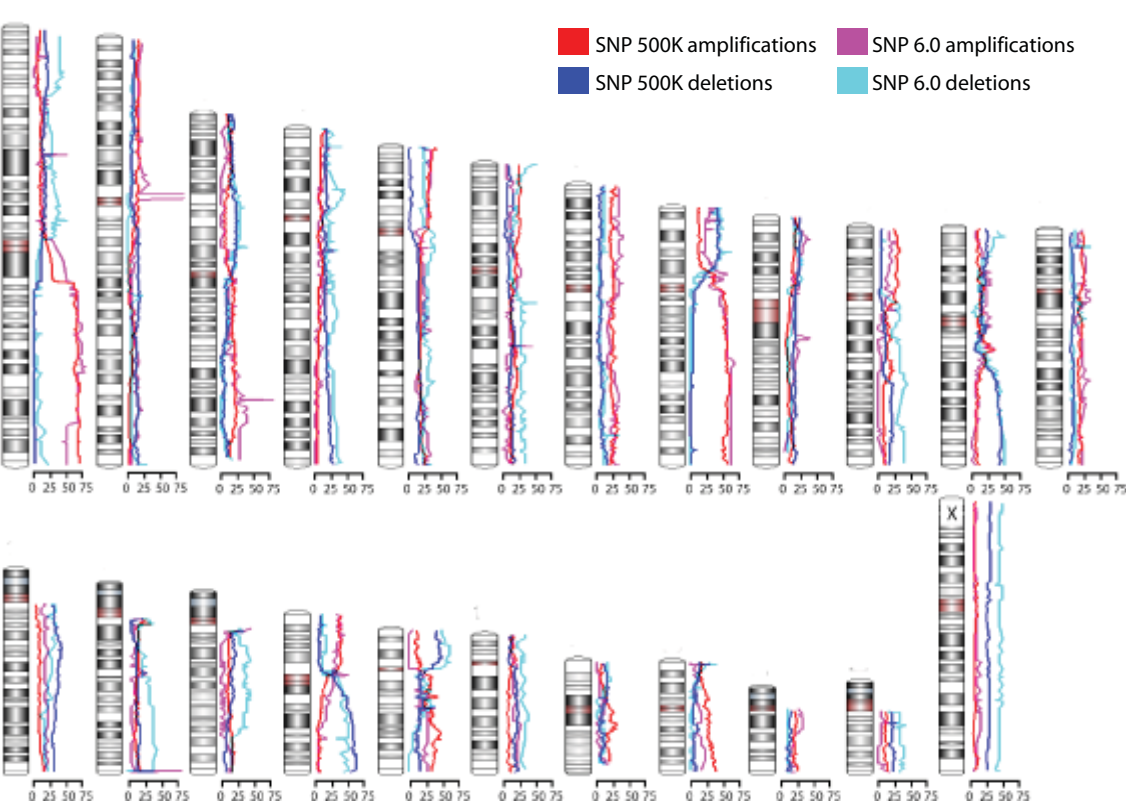

Supplement: Additional file 2: Figure S1. — Copy number variations observed in the 65 breast tumors showing the frequencies of genomic copy number gains and losses plotted according to their genomic localization. Blue lines correspond to allelic losses and red lines depict gains. Data from SNP 500 K and SNP 6.0 are displayed separately. Sixty percent of the tumors have amplification of chromosome 1q. A third of the samples have amplification of chromosome 5p, while about half of the tumors have loss of chromosome 8p from p.12 and beyond – frequently in combination with amplification of chromosome 8q, identified in about 60% of the tumors. Almost a third of the tumors show a high copy number amplification of the end of chromosome 8 from p.11.21 into the beginning of p.12 and approximately half of the tumors have loss of chromosome 11q from q.14.1 to the telomere. Finally, amplification of chromosome 16p is seen in almost half of the tumors and often in combination with loss of chromosome 16q, while loss of chromosome 17p is seen in about 40% of the tumors. [file 12885_2015_1035_MOESM2_ESM.pdf]

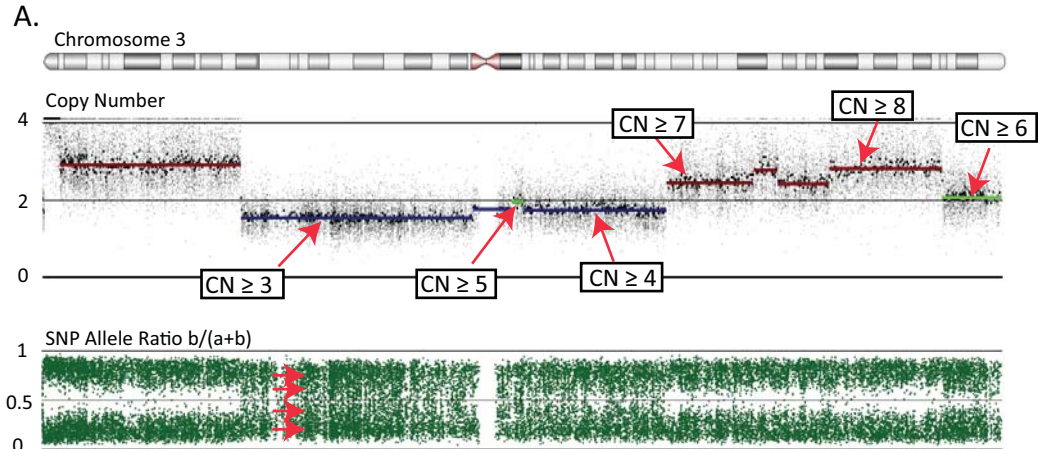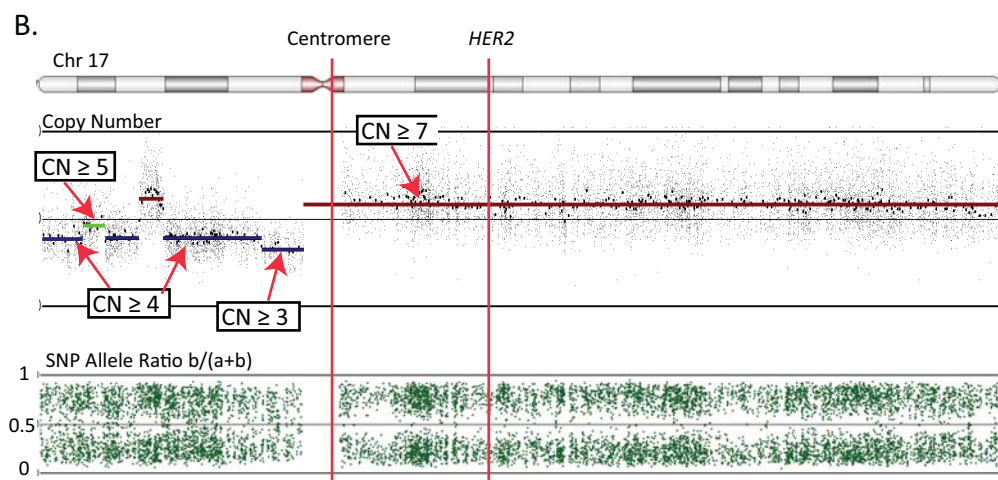

**C.** FISH with 3D rendering of confocal image stack

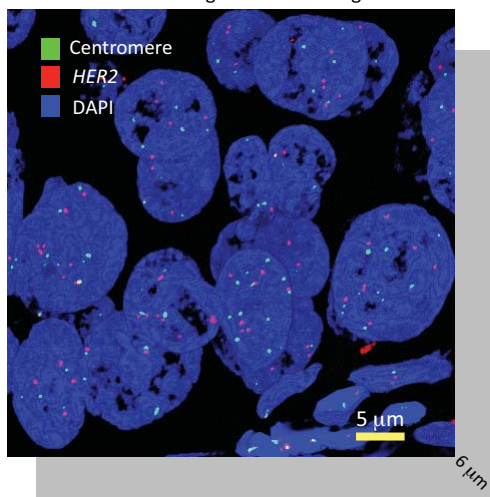

Supplement: Additional file 3: Figure S2. — Detection of polyploidy. (A) SNP and copy number data across chromosome 3 from tumor sample 45. The top panel displays the copy number probe intensity calls and the calculated copy number segments (in color). The lower panel displays the calculated SNP allele ratios of chromosome 3. The calculated segments have varying intensity values. The fragment with the lowest intensity value represents at least 3 copies because it exhibits allelic imbalance but still displays SNP heterozygosity (red arrows). Each fragment can be assigned an increasing copy number intensity, revealing that the predicted ‘copy number 2’ intensity corresponds to between 5 and 6 copies of DNA. (B) Subsequent examination of chromosome 17 shows that HER2 must be present in at least 7 copies. This sample also displays amplification of the centromere region (q-arm side) to the same extent as HER2, explaining why the FISH HER2/CEP17 ratio is 1.66. The vertical red line indicates the position of the centromere and HER2. (C) Representative image of a 3D-rendered model of a confocal image stack of a section from tumor sample 45 hybridized with HER2 (red) and CEP17 (green) probes. The image extends 6 μm down into the z-axis, corresponding to ~60–70% of the nucleus diameter. [file 12885_2015_1035_MOESM3_ESM.pdf]
